# A search filter for increasing the retrieval of animal studies in Embase

**DOI:** 10.1258/la.2011.011056

**Published:** 2011-10

**Authors:** Rob B M de Vries, Carlijn R Hooijmans, Alice Tillema, Marlies Leenaars, Merel Ritskes-Hoitinga

**Affiliations:** 1 3R Research Centre, Central Animal Laboratory, Radboud University Nijmegen Medical Centre, PO Box 9101, 6500 HB Nijmegen, The Netherlands; 2 Medical Library, Radboud University Nijmegen Medical Centre, Nijmegen, The Netherlands

**Keywords:** Three Rs, ethics and welfare alternatives, ethics and welfare supplements to animal research, search filter, systematic review

## Abstract

Collecting and analysing all available literature before starting a new animal experiment is important and it is indispensable when writing systematic reviews of animal research. In practice, finding all animal studies relevant to a specific research question turns out to be anything but simple. In order to facilitate this search process, we previously developed a search filter for retrieving animal studies in the most often used biomedical database, PubMed. It is a general requirement for systematic reviews, however, that at least two databases are searched. In this report, we therefore present a similar search filter for a second important database, namely Embase. We show that our filter retrieves more animal studies than (a combination of) the options currently available in Embase. Our search filters for PubMed and Embase therefore represent valuable tools for improving the quality of (systematic) reviews and thereby of new animal experiments.

Collecting and analysing all available literature before starting a new animal experiment is important and it is indispensable when writing a systematic review (SR) of animal research. An SR may be defined as a literature review focused on a single question which tries to identify, appraise, select and synthesize all available high-quality research evidence relevant to that question.^1^ SRs are already common in the field of clinical research. Even though clinical research is often based on preceding animal experiments, SRs of animal experiments, however, are still rather scarce.^2^ For several interrelated reasons, SRs of animal experiments should become standard practice as well^3^: (1) SRs will contribute to an improvement of the scientific quality of animal experiments; (2) they will lead to a reduction of the number of laboratory animals both absolutely, by preventing the unnecessary duplication of animal experiments, and relatively, by ensuring that the maximum amount of information is derived from the experiments that are carried out and (3) they are likely to increase patient safety, because SRs produce information relevant for judging the safety and efficacy of drugs that is not directly visible in the individual animal studies.^4^

Despite their importance, it is currently rather difficult to perform ‘high-quality’ SRs of animal research. This is partly because the methodological quality and the reporting of animal studies leave a lot to be desired,^5–7^ but also because it is anything but simple to find all animal studies relevant to a research question. Being as complete as possible is vital in case of SRs, since missing relevant papers may hamper the interpretation of the data and result in biased conclusions.^8^ In practice, finding all relevant studies turns out to be a challenge, particularly for scientists unfamiliar with using advanced search methods in large literature databases. An optimal search strategy pertaining to preclinical animal research typically consists of three components: strings of search terms related to (1) the disease of interest, (2) the intervention studied and (3) the animal (model) species used.^9^ In order to facilitate finding all relevant animal studies, in other words to optimize the third component, our group previously presented an easy-to-use search filter for PubMed,^10^ the database that contains the majority of medicine-related animal studies. Both in general and when combined with specific search strategies, this filter retrieves more animal studies than the option already present in PubMed, the *Limit: Animals*.

Given the importance of being complete, it is a general requirement for SRs that at least two databases are searched.^11^ The two databases that, aside from PubMed, are most relevant for identifying animal studies are Web of Science and Embase. Because Web of Science does not have a thesaurus and because using extensive search strategies including search filters is not yet feasible in this database, we decided to develop a search filter for Embase. Although there is considerable overlap between PubMed and Embase, the latter contains many journals that are not included in the former, notably European and pharmacological journals.

As Embase is a useful complement to PubMed, it is important to be able to identify all animal studies relevant to a particular research question in this database as well. Embase offers several options in order to perform a (more) specific search for animal experiments. First, there are a number of Emtree terms (i.e. thesaurus terms similar to PubMed's MeSH terms) that are more or less directly related to animal studies: ‘nonhuman’, ‘animal’, ‘animal experiment’, ‘experimental animal’ and ‘animal model’. The first two Emtree terms, however, are too broad, as the former includes plants/microoganisms and the latter not only pertains to vertebrates but also to invertebrates. In this paper and in our search filter, we follow the legal definition of animal experiment employed in most countries, according to which experiments on invertebrates are not considered animal experiments. The last three Emtree terms are more specific, but the assignment of these terms to papers turns out to be less than perfect: we have found quite a few papers that clearly report the results of animal experiments but to which none of these terms had been assigned.

Secondly, three limits are currently available: Animals, Animal Studies and Experimental Subjects/category heading Animals (with the option to select all species/groups of animals mentioned under this heading). The underlying search strategy for the first two limits is clear. The limit Animals is equivalent to the search string: *(exp animal/OR exp vertebrate/OR exp invertebrate/) NOT human/*; the limit Animal Studies to the string: *animal experiment/OR animal model/OR animal tissue/OR animal cell/*. A problem with the first limit is that, because of the ‘NOT human’-part, papers that report both animal experiments and studies in humans are excluded. The second string is problematic because the first half consists of Emtree terms the assignment of which is less than optimal (see above), while the second half refers to *in vitro* rather than *in vivo* studies. The category heading Animals is a very recent addition to the limit Experimental Subjects and we were unable to find out the exact search string behind it. Even though, compared with the other two limits, more records are retrieved by using this limit, there still appeared to be room for improvement.

We have therefore developed a search filter for Embase (under the OvidSP search platform; WoltersKluwer Health), similar to the one we designed for PubMed.^10^ Starting from this previously developed filter, we searched for Emtree terms equivalent to the MeSH terms in the PubMed filter and for related Emtree terms that did not correspond with MeSH terms but were relevant for the subject of animal experimentation.

In addition, a part to search for relevant terms in the titles and/or abstracts of records (a tiab-part) was added. Such a part was used in the PubMed filter in order to also retrieve the most recent papers, which have not yet received MeSH terms. Since in Embase all papers are indexed, a tiab-part would at first glance seem unnecessary. However, because of the deficiencies in the indexing process discussed in the previous paragraphs and given the importance of being as complete as possible, we added a tiab-part to our Embase filter as well. Compared with the tiab-part of the PubMed filter, a few minor modifications were made, e.g. (parts of) the scientific names of all the species included were added.

Our search filter for retrieving animal studies in Embase can be found in Supplement 1 (see http://la.rsmjournals.com/cgi/content/full/la.2011.011056/DC1). The number of records found by using this search filter and the limits Animals and Animal Studies are shown in Table 1. (Because we did not know the search string behind the limit Experimental Subjects/category heading Animals, we were unable to run this string and thereby determine how many records this limit retrieves on its own.) Application of the search filter resulted in many hits not found with (a combination of) the limit Animals and the limit Animal Studies. A random sample showed that a substantial number of these extra hits pertain to animal experiments.

**Table 1:** Results of searches in Embase using limits and our search filter (search date 23 May 2011)

Search	Search string	No. of records retrieved
1	Limit Animals	3,682,990
2	Limit Animal Studies	2,437,901
3	1 OR 2: combination of limits	4,149,787
4	Search filter: Emtree-part	4,256,629
5	Search filter: tiab-part	3,550,030
6	Whole search filter	4,850,933
7	6 NOT 3: extra records through search filter	1,010,142
8	Probiotics & pancreatitis & combination 3 limits	44
9	Probiotics & pancreatitis & search filter	70
10	Pregnancy & renal arteries & combination 3 limits	360
11	Pregnancy & renal arteries & search filter	395

The added value of the search filter becomes even more obvious when looking at examples of specific search strategies: we tested one strategy for finding animal experiments studying the effect of probiotics on acute pancreatitis and one for finding animal studies regarding the effect of pregnancy on the functional vascular properties of renal arteries (for full search strategies, see Supplement 2: http://la.rsmjournals.com/cgi/content/full/la.2011.011056/DC2). In case of the search strategy on probiotics and pancreatitis, the search filter led to 26 (59%) more records than a combination of the three limits available in Embase (Animals, Animal Studies and Experimental subjects/category heading Animals). Of these 26 hits, more than half were relevant to the subject, although these were mainly reviews. In case of the search for animal studies on the effects of pregnancy on renal arteries, 38 extra records (11%) were found through the search filter (the combination of limits yielded 3 records not found through the search filter, but none of these records pertained to animal studies.) These extra records contained 13 reviews discussing animal studies about this subject as well as three primary animal studies not found through application of the limits in Embase.

All in all, our search filter retrieves more animal studies than the options currently available in Embase. It is important to stress that the primary aim of the search filters is to make the search as complete as possible, i.e. the main objective is to ensure that all the relevant papers are found, rather than to avoid capturing potentially irrelevant papers. If the other components of the search strategy (related to disease and intervention) are properly selected, however, the total number of irrelevant papers retrieved will be minimal. Moreover, it is much easier to copy and paste our search filter in Embase, and save it for future use, than to combine the three limits. (Note that the filter should be copied into the search box using the basic search mode with the option ‘Include Related Terms’ disabled.) Because of the importance of completeness, particularly in the context of performing SRs, and because of its practical advantages, we therefore strongly recommend using our search filter rather than the limits in Embase.
